# Symptomatic Gallstones: Management Options for the 1990s

**DOI:** 10.1155/1991/41782

**Published:** 1991

**Authors:** J. Toouli

**Affiliations:** Gastrointestinal Surgical Unit Department of Surgery Flinders Medical Centre Bedford Park, S.A. Adelaide 5042 Australia


*HPB Surgery*, 1991, Vol. 4, pp 255–260
Reprints available directly from the publisher
Photocopying permitted by license only

© 1991 Harwood Academic Publishers GmbH
Printed in the United Kingdom

### SYMPTOMATIC GALLSTONES:

### Management Options For The 1990s

J. TOOULI
*Gastrointestinal Surgical Unit, Department of Surgery, Flinders Medical Centre, Bedford Park, S.A., 5042 Adelaide, Australia*

*(Received 7 March 1991)*

KEY WORDS: Gallstones, laparoscopic cholecystectomy, cholecystectomy, ESWL, dissolution, ultrasonography

### INTRODUCTION

At the recent annual meeting of the International Hepatobiliary and Pancreatic Association in Hong Kong the following panel was assembled with the aims of discussing the efficacy of the various methods of treatment available for patients with symptomatic gallstones. The panel comprised Drs S.K. Lam, G. Stevenson, T.K. Choi, G. Berci, E. Mack and F. Moody and it was Chaired by J. Toouli.

### INCIDENCE AND SYMPTOMS

Gallstones occur commonly in Western countries. It has been estimated that during normal life expectancy, 22% of men and 33% of women have gallstones, and that in 50% of cases these are symptomatic[1]. In the United States, for example, some 15 million people may have gallstones, the stones becoming symptomatic in some three million cases between the ages of 55–60 years. Half a million cholecystectomies are performed yearly to treat gallstones. The stones usually in Western patients are normally cholesterol stones or mixed stones. Black pigment stones occur less frequently. In Asia, cholesterol stones are much less common, and the predominant types are brown pigment and mixed stones. Brown pigment stones are amorphous, soft, and muddy, and thus tend to break up readily; an important feature when lithotripsy is being considered as a form of therapy. The male to female ratio of Chinese patients with gallstones is about 2 to 1 as opposed to the ratio of 1 to 3 generally seen in Western countries.

The major symptom produced by gallstones is pain. Dyspepsia, flatulence or nausea are symptoms which are not specific to gallstones. Available data indicate that treatment of gallstones should only be considered once symptoms develop[2,3].

Address correspondence to: James Toouli, Professor & Head of Unit, Gastrointestinal Surgical Unit, Department of Surgery, Flinders Medical Centre, Bedford Park, S.A., 5042 Adelaide, Australia

In patients with asymptomatic gallstones, only approximately 10–20% eventually develop symptoms.

### INVESTIGATION

Ultrasonography is the initial investigation used when cholelithiasis is suspected[4], it has a sensitivity and specificity which are both between 94–98%. Unlike oral cholecystography, which has comparable reliability, ultrasonography avoids exposure to radiation. Cholecystography should be used only when doubt remains after ultrasonography. Ultrasonography is useful in the diagnosis of acute cholecystitis in that oedema of the gallbladder wall may be detected. However, the sensitivity and specificity of this sign has yet to be precisely determined. If there is doubt about the diagnosis of cholecystitis, scintigraphy is used to determine cystic duct patency. An obstructed cystic duct supports the diagnosis of acute cholecystitis.

In patients being evaluated for extracorporeal shock wave lithotripsy (ESWL) or dissolution therapy the oral cholecystogram is used to determine cystic duct patency and size of the gallstones. CT scan evaluation of the gallstones has been used recently to detect rings of calcium in the stone[5]. Such stones are resistant to treatment by ESWL or dissolution.

Following pancreatitis ultrasonography is the most appropriate investigation to detect gallbladder stones. However, to determine whether stones are present in the bile duct, endoscopic cholangio pancreatography (ERCP) is the most accurate investigation. Ultrasonography has a low sensitivity for detecting stones in the bile duct.

### CHOLECYSTECTOMY

Cholecystectomy remains the "gold standard" for the treatment of gallstones. It has an operative mortality of less than 0.2%, a low morbidity and the majority of patients are cured of their disease[6]. A small percentage of patients may develop post-cholecystectomy biliary problems due to persistant or recurrent stone disease, iatrogenic injury or motility disorders affecting the sphincter of Oddi.

Once stones have passed from the gallbladder into the bile duct the treatment options depend on the patients age and the presence of associated medical conditions. In patients without a gallbladder the preferred treatment for gallstones in the bile duct is endoscopic sphincterotomy with extraction of the stones by a basket or balloon catheter. In patients with a gallbladder, choledochotomy at the time of cholecystectomy is a safe procedure with low morbidity[8]. However in elderly or infirm patients, endoscopic sphincterectomy is recommended to extract the bile duct stones leaving the gallbladder *in situ*. The overall incidence of problems arising from the retained gallbladder is approximately 15%; if the cystic duct is obstructed this incidence rises to around 50%[9].

### LAPAROSCOPIC CHOLECYSTECTOMY

Laparoscopic Cholecystectomy has created great interest since its recent introduction. Series with large numbers of patients are being reported from France[10], the

USA[11] and Britain[12]. What is intriguing is the fact that patients are ambulant on the day after the procedure, with rapid discharge from hospital and a return to normal activity, including work, within a week. On initial assessment there appear to be enormous advantages in carrying out cholecystectomy by laparoscopic means. Morbidity and mortality appear to be similar to that of open cholecystectomy[10]. The principles of the operation are identical to those for open surgery and precautions to avoid iatrogenic injury need to be observed meticulously. As with any new procedure, training is vital for surgeons embarking on this type of surgery. In the opinion of many hepatobiliary surgeons, the laparoscopic approach to the biliary tract will revolutionize surgery of the biliary tract. It is estimated that approximately 70% of patients with symptomatic gallstones can be treated in this way[12]. It is recommended at this stage that patients with acute cholecystitis should still be treated by open cholecystectomy. Similarly, patients who have previously undergone surgery to the upper abdomen should be excluded as adhesions may make laparoscopy hazardous. However future developments in laparoscopic techniques may reduce some of the contraindications and allow treatment of more patients with symptomatic gallstones.

### DISSOLUTION OF GALLSTONES

A variety of agents have been used to dissolve gallstones. The attraction of these agents for patients with symptomatic gallstones and their clinicians is the possibility of treating the stones without major intervention. However the fact that the gallbladder remains is often associated with significant recurrence of the gallstones and the need for repeated treatment.

Agents for oral dissolution include ursodiol (ursodeoxycholic acid, UDCA), chenodiol (chenodeoxycholic acid CDCA) and terpenes (mainly menthol). These agents can be used alone or as adjuvant therapy. They can only be used for the treatment of radiolucent cholesterol gallstones and require the presence of a patent cystic duct. CDCA acts by expanding the bile acid pool and inhibits cholesterol synthesis and secretion. Diarrhoea, which is dose related, affects approximately 50% of patients. CDCA leads to an increase in serum cholesterol as well as transient increase in serum amino transferases. The dose of CDCA is 15mg/kg/day and the efficacy of dissolution is approximately 30–40%[13]. The duration of oral bile acid therapy for dissolution is 1–2 years and the cost is approximately $US1.00/day.

UDCA acts by inhibiting cholesterol secretion in bile and reduces gut cholesterol absorption. It promotes non-micellar mechanisms of cholesterol solubilization and has practically no side effects. The dosage of UDCA is 10mg/kg/day and the efficacy of dissolution for cholesterol gallstones is 50–80%. Duration of therapy is approximately 1–2 years and the cost of the medication is $US3–4.00/day[13].

Contact solvents used to dissolve cholesterol gallstones include mono-octanoin, methyltert-butyl ether (MTBE), D-limonene, GS-100, and EDTA. Mono-octanoin has been used more extensively, mainly for dissolving stones in the bile duct. Side effects include nausea, vomiting, pain and diarrhoea. The efficacy for dissolution of bile duct stones is 50–80% following continuous infusion into the bile duct for up to 1 week.

MTBE is an experimental agent which is mainly employed for cholesterol stones within the gallbladder. It is explosive and malodorous and it causes nausea and overflow sedation, as well as duodenitis. The duration of treatment for gallstone dissolution is 1–3 days and efficacy is 90–100%. EDTA-bile acid mixtures have been used experimentally for brown pigment stones. The disadvantages are again a slow dissolution rate and an adjuvant agent or technique is needed for successful outcome[14].

The major disadvantage of all dissolution agents is the recurrence rate which for CDCA and UDCA is recorded at 50% at 5 years. However in patients deemed to be at major risk following more invasive procedures, dissolution therapy provides a minimally invasive avenue for therapy. The indications to use mono-octanoin to treat bile duct stones have diminished with the availability of endoscopic and percutaneous methods of stone extraction. However occasionally it may be used as an adjuvant to the extraction technique in order to soften or reduce the size of a large stone so that it might more easily be removed[15].

### EXTRACORPOREAL SHOCK WAVE LITHOTRIPSY

Non interventional treatment of gallstones appeared to become reality with the first reports of extracorporeal shock wave lithotripsy (ESWL) in the treatment of symptomatic gallstones[16]. Results from a trial conducted in Munich using a water bath spark-gap acoustic generator reported efficacy of approximately 90% in appropriate patients maintained on chenodiol and ursodiol for one year after lithotripsy. On the basis of this experience trials were initiated using three types of acoustical wave generators; spark-gap, electromagnetic and piezo electric. The spark gap generator has a broader acoustical focus at the level of the target zone and therefore has a greater success at shattering the stones. However it requires analgesia and sedation during treatments. The electromagnetic and piezo electric machines offer relatively pain-free treatment but multiple treatments because of their small focal zone. All of the techniques require adjuvant bile salt therapy for approximately one year to obtain stone clearance.

Experience in subsequent trials, conducted mainly in the USA have provided the following principles. ESWL is safe and well tolerated. Selection and targetting are essential for success. The fragmentation rate is approximately 90% in patients with non-calcified stones. Approximately 40% of patients have biliary colic in the early weeks after treatment. Thirty per cent will have stone fragments of a size that will benefit from retreatment. Approximately 10% of patients require cholecystectomy for continuing symptoms and all patients require bile salt therapy to achieve stone clearance. The overall success rate for stone clearance at 6 months is between 42 and 69%, but this may improve up to 90% at one year[19]. A major drawback is the recurrence rate after successful dissolution. Early reports suggest a recurrence rate of stones of approximately 10% per year and is similar to the recurrence rate reported after either UDCA or CDCA therapy[21].

### RECOMMENDATIONS

The panel concluded that symptomatic gallstones, as opposed to asymptomatic stones, warrant treatment. The initial investigation to demonstrate gallstones is ultrasonography and in the majority of patients, this investigation suffices prior to treatment. Cholecystectomy is the most efficacious therapy for symptomatic gallstones in most patients. With the evolution of laparoscopic cholecystectomy and its attributes, this technique is poised to become the gold standard for the treatment of gallstones. In patients with stones in the bile duct presenting some time after cholecystectomy and demonstrated by ERCP, extraction after endoscopic sphincterotomy provides the best approach. All other techniques for the management of gallstones are either inferior to the above or are experimental and recommended only for specific clinical situations which do not allow the use of the above techniques. Dissolution therapy using oral agents or contact solutions are confined to patients with medical disorders which make the above procedures hazardous. ESWL is used as an aid to dissolution. However its overall efficacy requires further study. The major drawback for both dissolution and ESWL is the high recurrence of gallstones whereas cholecystectomy by laparoscopic means or open surgery deals with the diseased gallbladder permanently.

### *References*

1. McSherry, C.K., Ferstenberg, H., Calhoun, W.F., Lahmana, E. and Virshup, M. (1985) The natural history of diagnosed gallstone disease in symptomatic and asymptomatic patients. *Ann. Surg.*, **202**, 59–63
2. Ransohoff, D.F., Gracie, W.A., Wolfenson, L.B. and Neuhauser, D. (1983) Prophylactic cholecystectomy or expectant management for silent gallstones. A decision analysis to assess survival. *Annals of Internal Medicine*, **99**, 199–204
3. Rome Group for the epidemiology and prevention of cholelithiasis (GREPCO) (1984) Prevalence of gallstones disease in an Italian adult female population. *American Journal of Epidemiology*, **119**, 796–805
4. Bartrum, R.J. Jr, Crow, H.C. and Foote, S.R. (1977) Ultrasonic and radiographic cholecystography. *New England Journal of Medicine*, **296**, 538–541
5. Brakel, J., Lameris, J.S., Nijs, H.G.T., Terpstra, O.T., Steen, G. and Blijenberg, B.C. (1990) Predicting gallstone composition with CTL: in vivo and in vitro analysis. *Radiology*, **174**, 337–341
6. McSherry, C.K. and Glenn, F. (1980) The incidence and causes of death following surgery for non-malignant biliary tract disease. *Annals of Surgery*, **191**, 271–275
7. Cotton, P.B. (1984) Endoscopic management of bile duct disorders; (apples and oranges). *Gut*, **25**, 587–597
8. Worthley, C.S., Watts, J.McK. and Toouli, J. (1989) Common duct exploration or endoscopic sphincterotomy for choledocholithiasis. *ANZ Journal of Surgery*, **59**, 209–216
9. Worthley, C.S. and Toouli, J. (1989) Gallbladder non-filling: an indication for cholecystectomy following endoscopic sphincterotomy. *Br. J. Surg.*, **75**, 796–798
10. Dubois, F., Berthelot, G. and Levard, H. (1989) Cholecystectomie par Coelioscopie. *Presse Med.*, **18**, 980–982
11. Reddick, E.J. and Olsen, D.O. (1989) Laparoscopic laser cholecystectomy. A comparison with mini-lap cholecystectomy. *Surg. Endosc.*, **3**, 131–133
12. Cushieri, A., Berci, G. and McSherry, C.K. (1990) Laparoscopic Cholecystectomy. *Amer. J. Surg.*, **159**, 273
13. Hofmann, A.F. (1989) Medical dissolution of gallstones by oral bile acid therapy. *Amer. J. Surg.*, **158**, 198–204
14. Mack, E. (1986) Chemical dissolution of biliary stones. *Contemp. Surg.*, **28**, 20–23
15. Mack, E. (1990) Role of surgery in the management of gallstones. *Seminars in Liver Disease*, **10**, 222–231
16. Sauerbruch, T., Delius, M., Paumgartner, G. *et al.* (1986) Fragmentation of gallstones by extracorporeal shock waves. *N. Eng. J. Med.*, **314**, 822–828
17. Sackmann, M., Delius, M., Sauerbruch T. *et al.* (1988) Shock wave lithotripsy of gallbladder stones. *N. Engl. J., Med.*, **318**, 393–397
18. Albert, M.B. and Fromm, H. (1990) US Multicenter study of the safety and efficacy in treating gallstones with Piezoelectric lithotripsy. *Gastroenterology*, **98**, Part 2 A564

19. Burnett, D., Ertan, A. and Jones, R. (1989) Use of external shockwave lithotripsy and adjuvant ursodiol for treatment of radiolucent gallstones. A national multicenter study. *Dig. Dis. Sci.*, **34**, 1011–1015
20. Darzi, A., El-Sayed, E., Tanner, W.A. and Keane, F.B.V. (1990) Gallstone recurrence after successful shock wave lithotripsy. *Gastroenterology*, **98**, Part 2 A580
21. Villanova, N., Bazzoli, F., Taroni, F., Frabboni, R., Mazzella, G., Fresti, D., Barbara, L. and Roda, E. (1989) Gallstone recurrence after successful bile acid treatment. A 12 year follow-up study and evaluation of longterm post-dissolution treatment. *Gastroenterology*, **97**, 726–731

*(On invitation by S. Bengmark, received 7 March 1991)*

